# Authors reply to: Interpreting whole‐body carbohydrate oxidation and ‘oxidation efficiency’ in carbohydrate supplement studies

**DOI:** 10.1113/EP093858

**Published:** 2026-04-19

**Authors:** Ewan Dean, Ash Osbrone, Daren Subar, Paul Hendrickse, Christopher J. Gaffney

**Affiliations:** ^1^ Lancaster University Medical School Lancaster University Lancaster UK; ^2^ BRIDGES Research Group, Department of Surgery East Lancashire Hospitals NHS Trust Blackburn UK

**Keywords:** carbohydrate metabolism, exercise, nutrition, oxidation, supplements

1

We read with interest the comments of Dr Pettersson on our recent work on the comparative analysis of three commercially available carbohydrate supplements (Dean et al, 2025). We thank the author for their interest in our work and for his detailed and constructive comments. We recognise the importance of aligning methodological capability with interpretation when examining substrate metabolism, and we welcome the opportunity to clarify several key points.

A central issue raised concerns the use of whole‐body indirect calorimetry and its inability to distinguish between exogenous and endogenous carbohydrate oxidation (Davies, 2020; Gonzalez and King, 2022). We fully acknowledge throughout the paper that, without stable‐isotope tracer methodology, indirect calorimetry quantifies total carbohydrate oxidation and does not differentiate between substrate sources (Jeukendrup and Wallis, 2005), as further highlighted in the limitations section of our paper.

The primary aim of this study was to compare postprandial glucose and metabolic responses to three commercially available carbohydrate formulations at rest and during high‐intensity exercise, and to explore their potential influence on repeated sprint performance. Thus, our focus was not on directly quantifying exogenous carbohydrate oxidation, but on characterising whole‐body metabolic responses under controlled feeding conditions.

In the exercise trials, the provision of a standardised pre‐exercise meal of 196 kcal, consisting of 64% carbohydrate (27.0 g, of which sugars 11.3 g), 18% fat (7.6 g) and 8% protein (3.6 g), with 2.7 g fibre and 0.36 g salt, may have minimised between‐visit variability in basal hepatic glucose production. Therefore, although indirect calorimetry reflects whole‐body carbohydrate oxidation and does not distinguish between exogenous and endogenous sources, any differences observed between conditions are likely attributable to the metabolic effects of the ingested carbohydrate formulations. This may include differences in the suppression of hepatic glucose production, such that variation in endogenous glucose contributions forms part of the physiological response to each product, rather than a confounding factor. In contrast, the resting OGTT trials were conducted following a self‐replicated breakfast, which, although not quantitatively recorded, was standardised within participants across visits, as shown in Figure 2a (Dean et al, 2025), where there was no difference in resting glucose (GF‐Bar; 4.50 ± 0.67 mmol/L, GF‐Gel; 4.50 ± 0.53 mmol/L, MD‐Gel; 4.41 ± 0.39 mmol/L, *P* = 0.7). This approach preserves ecological validity and reflects typical pre‐prandial conditions, while maintaining consistency at the individual level.

Regarding oxidation efficiency, we acknowledge the interpretive challenges surrounding this metric. Carbohydrate oxidation efficiency, calculated as total carbohydrate oxidised relative to the ingested dose, serves as a normalised index of whole‐body metabolic response. Further, the absence of a significant product × time interaction for the data on carbohydrate oxidation per minute indicates that differences across individual time points are exploratory. Primary conclusions are based on overall product effects over the measurement period. It is important to note that the carbohydrate oxidation data cannot distinguish between exogenous and endogenous sources of carbohydrate oxidation and should not be interpreted as a direct measure of the proportion of ingested carbohydrate specifically oxidised from exogenous sources. We acknowledge throughout and explicitly state in the limitations section that, without isotopic labelling, this metric cannot distinguish between exogenous and endogenous carbohydrate sources (Davies, 2020; Gonzalez and King, 2022; Wrench et al, 2024). Values approaching 100% reflect combined contributions from exogenous intake, hepatic glucose output and glycogen mobilisation. Therefore, this metric should be understood as providing a relative comparison of whole‐body responses, rather than a direct estimate of exogenous carbohydrate utilisation.

The author raises valid concerns about the mechanistic interpretation of the observed differences in fat oxidation and their implications for performance. We agree that the absolute differences noted during the resting protocol are modest. These findings likely reflect subtle shifts in whole‐body substrate utilisation rather than functionally significant changes in energy provision. This is further evidenced by the absence of differences in sprint performance across conditions, which aligns with the physiological demands of short‐duration, high‐intensity exercise that predominantly utilises phosphocreatine and intramuscular glycogen (Vigh‐Larsen et al, 2024), thereby limiting the contribution of circulating or exogenous carbohydrates to performance. Furthermore, only a subset of participants completed both the OGTT and sprint trials, limiting direct linkage between resting metabolism and performance. Mechanistic interpretations connecting these resting differences to ergogenic potential should therefore be made with caution. As such, any subtle shifts in substrate utilisation observed during the OGTT could have different implications under endurance conditions, although this remains speculative in the absence of direct evidence.

While the present findings did not translate to improved performance during short‐duration, high‐intensity exercise, it is important to note that even relatively small carbohydrate intakes can influence performance in more endurance‐based exercise modalities. Indeed, carbohydrate mouth rinsing has also been shown to elicit a positive effect on endurance performance (Jeukendrup, 2014).

In summary, our findings demonstrate differences in whole‐body metabolic responses to commercially available carbohydrate formulations under the specific experimental conditions employed. However, they do not provide a direct assessment of exogenous carbohydrate oxidation or definitive evidence of product‐specific metabolic superiority, which we acknowledge. We agree that future studies incorporating stable‐isotope tracer methodology, alongside study designs that investigate specific mechanistic hypotheses, would provide further insights into exogenous carbohydrate utilisation and its relevance to performance.

## AUTHOR CONTRIBUTIONS

All authors have read and approved the final version of this manuscript and agree to be accountable for all aspects of the work in ensuring that questions related to the accuracy or integrity of any part of the work are appropriately investigated and resolved. All persons designated as authors qualify for authorship, and all those who qualify for authorship are listed.

## CONFLICT OF INTEREST

C.G. is a former editor for *The Journal of Physiology*.
